# Bibliographical Notices

**Published:** 1847-11

**Authors:** 


### BIBLIOGRAPHICAL NOTICES.

*Criticisms and Controversies relating to the Nervous and Muscular Systems.* By BENNET DOWLER M. D., of New Orleans. Reprinted from the New Orleans Medical and Surgical Journal, September, 1847.

The above is the title of a pamphlet of 70 pages, in which Dr. Dowler critices the criticisms of Dr. Marshall Hall, and some British reviewers, excited by certain experimental observations of Dr. D., relating to the muscular and nervous systems. These observations (published some two years ago, and an analysis of them presented to the readers of this Journal) were of a highly interesting character, and of not a little scientific importance. In our comments at the time of their publication, we took occasion to express regrets, that Dr. Dowler had not confined himself to a simple enunciation of facts and deductions, instead of indulging a strain of sarcasm, illy comporting, in our estimation, with the subject and occasion. But this fault of style evidently arises from a peculiarity of mental constitution, which cannot refrain from commingling the ludicrous with the gravity due to philosophical disquisitions. It denotes, in this instance, certainly, no malignity, nor ill humor, but a tendency to the comic which cannot be resisted. The facts reported by Dr. D., as our readers will recollect, appeared to militate against, and in the reporter's opinion, completely overturn the doctrines mantained by, and, to some extent, originating with Dr. Hall. Dr. Hall, too, appears to have some mental peculiarities. He seems to live in a perpetual state of excessive anxiety lest the professional public, and posterity, shall fail to do ample justice to the importance of his doctrines. Hence, he is constantly reiterating them in new publications, and seems to think it unsafe to leave the subject long, for fear that the reflex theory, or, at least its paternity will be entirely forgotten. He is also amazingly testy if any one ventures to express a surmise that Physiology, Pathology and Therapeutics contain aught in addition to the doctrine of reflex nervous action. We do not purpose here to enter into any discussion of the merits of this doctrine, or the claims of Dr. Hall as a discoverer of scientific truths. We will only say, that, with much that is new and useful a considerable portion of the doctrine consists of old things under new names, and a considerable portion is by no means fully established. Our present concern is with the mutual relations of Drs. Hall and Dowler, as a matter of medical gossip. Dr. Hall takes umbrage at tbe contributions of Dr. Dowler, and sends a short note to the editor of the New York Journal of Medicine, informing the Editor, and the American Medical Public, that the poor reflex doctrine had suffered many attacks, but, none to compare with that by Dr. Dowler. Dr. D. imputed to it a tendency to materialism; and this, Dr. H. denounces as "cowardice and calumny." The Anathema of Dr. H., together with some criticisms by the Medico-chirurgical review, has called out Dr. D., in the pamphlet before us. Altogether, the rejoinder is a "psycological phenomenon." Logic, learning, ridicule, humor, scientific facts, are thrown together in heterogenous combination.

It is a kind of mosaic production, which rhetorical ingenuity would find it difficult, if not impossible to imitate, were the trial to be made. It is *sui generis*. The author is evidently a man of scholastic and literary attainments, and, as a writer, has a wonderful amplitude of diction. But he is moreover an observer, an original thinker, and brings to the investigations of science an enterprising, inventive disposition, which is not content with travelling beaten paths, but delights to explore unoccupied territories. The sensitiveness and irritability of genius are to be added, to complete the "charcoal sketch" drawn from a perusal of his writings. We make this portrait without any personal acquaintance with the original, and with the knowledge that it will come under his own eye. Whether we do him less, or more than justice, we have no fears that himself and friends will not excuse the freedom with which, in in this instance, we have indulged in personalities. If we are not mistaken, Dr. D. possesses talents eminently fitting him for the field of labor which he has selected, viz., original investigations. He has already developed novel and interesting facts; he announces that he has more in reserve, and we trust, and believe, that, if life be spared, he will confer benefits on science, and win honor for himself We must express, therefore, our repugnance to see him wasting any portion of his time and energies upon controversial discussions. If he will heed the suggestion of one who is anxious to aid in heralding the results of his scientific labors, he will confine his attention to the latter, and leave the vexatious task of removing prejudices, and refuting illiberal criticisms, to those who cannot be more usefully employed.

---

*Lectures on Subjects Connected with Clinical Medicine.* By P. M. Latham, M. D., Fellow of the Royal College of Physicians, and Physician to St. Bartholomew's Hospital; second edition. Philadelphia; Barrington & Haswell; 8 vol., pp. 158.

This work contains fifteen lectures, the first half relating to a variety of subjects connected with the prosecution of Clinical studies; the last half devoted, mainly, to the Auscultatory signs of Pulmonary diseases.

The style of the lectures is eminently familiar and colloquial.—The book reads precisely as we may imagine the author talks; and we fancy that we can form a very clear apprehension of the manner in which he talks, by reading the book.

Dr. Latham's writings possess for ourselves a peculiar recommendation. In so far as he is pictured upon the mental retina while we are perusing his discourses, we are forcibly reminded of a distinguished American teacher, revered by all who were privileged to enjoy his instructions. We refer to James Jackson, M. D., formerly Professor of Medicine in the Massachusetts Medical College. Many who, like ourselves, can recall the learning, zeal, and truth-loving spirit which pervaded the lectures of that distinguished Professor, (and all who have heard his lectures will never cease to remember them as invested with these qualities,) will not construe the comparison into complimentary allusion to him, but will consider it the warmest eulogium which can be bestowed on the work before us.

In the foregoing remarks, the reader will observe we speak of Dr. Latham's writings in general. We have, as yet, examined only the first half of the present volume. There is much contained in the first eight lectures, which, for the student and practitioner, (and the latter term should involve the former) is both interesting and profitable ; but the subjects treated of, and the manner of treating them, preclude analysis. We may find in the portion allotted to Auscultatory signs, observations which we may deem important hereafter to present to our readers. In the meantime, we commend the book, not as superseding systematic works on similar subjects, but of containing valuable matter presented in a highly agreeable manner.

In concluding this brief notice, lest we may not conclude to review the latter half of the work, we beg leave to quote a few remarks from the preface. The reader who may recollect some views which we some time ago submitted under the heading of "The study of Auscultatory without a master," will perceive by the quotation which follows, that similar views are entertained by one who is confessedly among the most eminent Auscultators now living:—

" Auscultation is capable, I have thought, of being greatly simplified for practical purposes. At all events, unless it be so it can never be successfully taught ; the knowledge derived from it must be confined to a few physicians of hospitals, and the profession at large can never expect much benefit for its use.

"Whether I have succeeded in accomplishing what I think so desirable, and have cleared Auscultation of its mystery in any degree, others must judge. But thus much I can safely say, that the intelligent student, by attending to the few characetristic sounds which I have pointed out, and taking pains to understand their import, and guarding himself against over-refinement, is able in a few weeks to discern the leading truths connected with Auscultation, and in a few months to use it and trust it as his safest guide in the diagnosis of pulmonary diseases."

---

*A practical treatise on the Diseases of Children.* By D. Francis Condie, M. D., Secretary of the College of Physicians, Member of the American Philosophical Society, &c. Second edition. revised and augmented. Philadelphia: Lea and Blanchard, 1847. 8 vo. pp. 657.

We are somewhat dilatory in noticing the second edition of the above valuable treatise. The author has manifested his appreciation of the success of the first edition in a way which is most creditable to him, and will be most satisfactory to the Medical Public, viz., by his efforts to render the second edition still more valuable and meritorious. He states in the preface that "every part of the work has been subjected to a careful revision; several portions have been rewritten comprising all the more important facts, in reference to the nature, diagnosis, and treatment of the diseases of infancy and childhood, that have been developed since the appearance of the first edition." We cordially join with the medical press generally in recommending the work as an excellent practical guide in the treatment of infantile diseases. It is an *American book* which reflects credit on American Medical Literature.

---

*Outlines of the Veins and Lymphatics, with Short Descriptions. Designed for the use of Medical Students.* By John Neil, M.D., Demonstrator of Anatomy in the University of Pennsylvania, &c. Philadelphia : Edward Barrington and George D. Haswell. 1847. 8vo. pp. 29.

This little work is on the same plan with the two which have been already published, on the arteries and veins. It is calculated to aid very materially in acquiring knowledge of the anatomical systems which it is intended to illustrate ; and medical students, especially, will do well to obtain it.

*The American Medical Almanac for* 1848; Philadelphia, Lindsay & Blakeston; 16mo; pp 224.

This is really a very useful little work for the Physician and Medical Student. In addition to an Almanac, it contains circulars of all the Medical Schools in the United States; the code of Medicals Ethics adopted by the American Medical Convention; an account of the poisons commonly used for self-destruction, or murderous purposes, or by accident, with the symptoms and treatment; a digest of facts relating to Etherization, and a variety of miscellaneous matter. Altogether it makes a very good investment for a couple of shillings or so.

---

*Wood's Quarterly Retrospect of American and Foreign Practical Medicine and Surgery.* New-York: Richard and George S. Wood No 261 Pearl St. 8vo. pp. 64.

This quarterly periodical is on the plan of *Braithwaite's Retrospect*, and *Ranking's Abstract*—publications which have acquired an extensive circulation, and are deservedly much esteemed. The object of an American work on the same plan is to do more ample justice to American periodical literature, than the English works above named have done. Owing to the difficulty and expense attending the transmission of periodicals, the edition of those works do not have access to the greater portion of the Journals of this country, We think our friends, the Woods, have done a good thing in undertaking this enterprise, and we trust it will prove advantageous to them as well as to the profession. It is furnished gratuitously to the subscribers of the Medico-chirurgical Review, and to others for one dollar *per annum.* We must concur with some of our contemporaries who have expressed a desire to see the work under an improved typographical aspect. We gladly welcome any effort to promote the progress of *American Medical Literature*, and we therefore cordially bid this work *God speed.*

---

*Work on General Pathology.* By Prof. Chomel.—Wm. D. Ticknor and Co., of Boston, will shortly publish a translation of the third edition of Prof. Chomel's work entitled "Elements of General Pathology." It will be very acceptable to the Medical Profession of this country.

*New work on Obstetrics and the diseases of women and children.*—Dr. Jno. Evans, Prof. in Rush Medical College, announces that he has a work on the above subject in preparation, and solicits from western physicians the results of their observations upon the modifications of diseases peculiar to women and children in their respective districts.

---

*Western Lancet.*—This Journal is hereafter to be published at Cincinnati, Ohio, the Editor, Dr. Lawson, having removed from Lexington, Ky., to be connected with the Medical College of Ohio.

---

*The Annalist.*—This Journal has entered on the second year of its existence, and is now issued by the well known Medical Publishers, *the Woods*, to whose care communications, &c., should be addressed. There are few Journals which we greet with more pleasure than the Annalist. Dr. Roberts is eminently fitted for editorial duties, and devotes himself to the labors which these duties involve, with the zeal and energy which an ardent love for the profession alone can inspire. We are gratified to learn that his services are appreciated, and heartily wish his journal lengthened and increasing prosperity.

---

Two new Journals have lately been added to the list of American Medical Periodicals. One of these is entitled "*New Jersey Medical Reporter, and Transactions of the New Jersey Medical Society. Edited by Joseph Parrish, M. D.*. It is published at Burlington, 84 pages octavo, at two dollars per annum.

The other is a monthly of 44 octavo pages, subscription price two dollars per annum, issued at Memphis, Tennesee, and edited by James Conquest Cross, M. D., Prof. of Institutes, &c., in the Memphis Medical College; assisted by his colleagues. Its title is the *South-western Medical Advocate.*

We cordially extend the hand of fellowship to these, and all other aspirants for the *l bors* and the *rewards* of Medical Journalism, trusting that their zeal will sustain them beyond the period when editorial duties cease to be attractive from their novelty.

---

*Chelius's System of Surgery,* has been received and will be reviewed in our next number.

